# Thymic Carcinoma Presenting as a Mediastinal Mass Resembling a Cardiac Tumor

**DOI:** 10.7759/cureus.61455

**Published:** 2024-05-31

**Authors:** Shriya Doreswamy, Sakshi Mishra, Tejaswi Venigalla, Zahraa Al Turaihi, Supriya Sekhar

**Affiliations:** 1 Internal Medicine, Jefferson Einstein Montgomery Hospital, East Norriton, USA; 2 Otolaryngology - Head and Neck Surgery, Vydehi Institute of Medical Sciences and Research Centre, Bangalore, IND; 3 Internal Medicine, Einstein Medical Center Montgomery, East Norriton, USA; 4 Radiology, Einstein Medical Center Montgomery, East Norriton, USA

**Keywords:** y90 treatment, transarterial radioembolization, cancer chemotherapy, palliative radiation therapy, malignant thymoma, immunohistochemistry of squamous cell thymic carcinoma, high grade thymic squamous cell carcinoma

## Abstract

Thymoma and thymic carcinomas are a few of the rarest malignancies seen in humankind. They are mostly seen in the Asian population, many of which are reported in the Southeast Asia region like Japan, China, Vietnam, etc. They usually can be a sequela of other underlying conditions such as myasthenia gravis or some unknown mutations that express later in life.

Our patient is a young 41-year-male, a healthy and active individual who presented for evaluation of acute shortness of breath, two months after recovering from SARS-CoV-19 infection. His shortness of breath progressed while on oxygen and diuretics, a Point of Care Ultrasound (POCUS) showed cardiac tamponade and moderate pleural effusion. A Computerized Tomographic (CT) scan of the chest/abdomen/pelvis showed cardiomegaly, pleural effusion, and a mass abutting the heart. A pericardiocentesis revealed malignant cells. Thymic carcinoma was confirmed with a core biopsy and the patient was initiated on treatment rapidly to help improve symptoms and contain the growing mass.

## Introduction

The thymus, a lymph gland located in the center of the chest within the anterior mediastinum, plays a crucial role in immune development during fetal stages, infancy, and childhood before becoming vestigial. Tumors from this gland range from the less aggressive thymoma to the more aggressive thymic carcinoma [1]. Thymomas, thymic carcinomas, and thymic neuroendocrine tumors are collectively referred to as epithelial tumors. These are derived from epithelial cells [2]. Their incidence is relatively rare, at 0.15 per 100,000 individuals [3]. The invasiveness of its capsule gauges the tumor’s aggressiveness. While imaging techniques like computed tomography (CT) and magnetic resonance imaging (MRI) aid in characterizing these tumors, histopathological examination remains the gold standard for definitive diagnosis [4]. Treatment options, particularly for unresectable tumors, include chemotherapy and radiation therapy [5]. Thymomas and thymic carcinomas, carcinomas are known for their radiosensitivity, and present challenges in determining optimal radiation doses due to their rarity and indolent nature [6,7]. 

## Case presentation

A 41-year-old Asian man, non-smoker and non-drinker, presented to the emergency department (ED) with shortness of breath. He was an active ice hockey player and in overall good health. He required only an annual physical examination with his primary care physician. He had recovered from COVID-19 in June 2022 but had been experiencing progressive shortness of breath, preventing him from resuming sports and even lying flat. Upon arrival at the ED, he was put on 6 liters of oxygen via nasal cannula initially and then switched to a non-rebreather mask due to his breathing difficulties. His initial laboratory workup results are detailed in Table 1. 

**Table 1:** Laboratory investigations on presentation at the emergency department

Analyte	Patient Value
Sodium	128 mmol/L
Potassium	5.4 mmol/L
Bicarbonate	16 mmol/L
Serum Urea Nitrogen	33 mg/dL
Creatinine	1.24 mg/dL
Anion Gap	18
Phosphorus	6.2 mg/dL
Lactic Acid	10.78 mmol/L
Alkaline Phosphatase	153 U/L
Total Bilirubin	1.4 mg/dL
Direct Bilirubin	0.8 mg/dL
Alanine Aminotransferase (ALT)	1241 U/L
Aspartate Aminotransferase (AST)	1613 U/L
White Blood Cell (WBC) Count	17.8 x10^3/µL
Hemoglobin	12.8 g/dL
Red Cell Distribution Width (RDW)	18.6%
Platelets	624 x10^3/µL
Partial Thromboplastin Time (PTT)	29.1 seconds
Prothrombin Time (PT)	32.9 seconds
International Normalized Ratio (INR)	2.8

**Figure 1:**
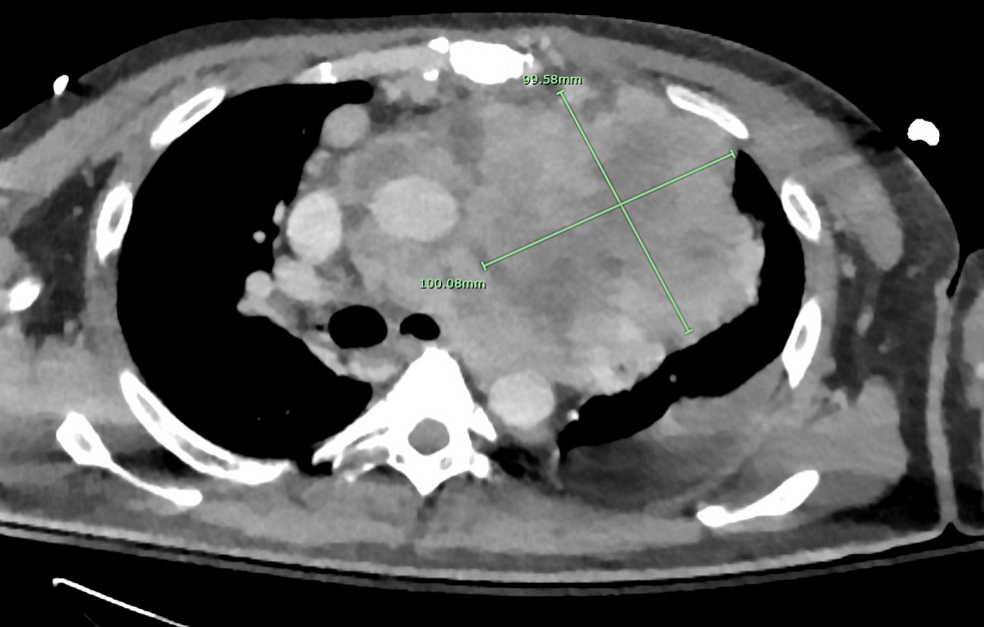
CT scan of the chest with IV contrast showing the mass at diagnosis.

An initial chest x-ray (CXR) revealed a large lobulated left mediastinal mass obscuring the left cardiac border, right paratracheal mediastinal widening, moderate cardiac enlargement, a small pleural effusion, and left lower lobe atelectasis. A subsequent point-of-care ultrasound in the ED detected a significant cardiac tamponade. A CT scan of the chest, abdomen, and pelvis further showed a dominant necrotic mass within the left anterior mediastinum, exerting pressure on adjacent vasculature and causing rightward deviation. The mass measured 9.9 cm x 10 cm x 12 cm (Figure 1). Additional findings included mediastinal lymphadenopathy, scattered pulmonary nodules, and prominent supraclavicular lymph nodes suggestive of lymphomatous disease. Severe cardiomegaly and moderate ascites were also observed. Ultrasound of the liver indicated mild hepatomegaly and hepatic steatosis. Furthermore, cholelithiasis and mild nonspecific gallbladder wall thickening were noted, though Murphy’s sign and biliary dilation were negative during ultrasound imaging. Radiologists recommended tissue sampling for further evaluation of the mediastinal mass. 

**Figure 2:**
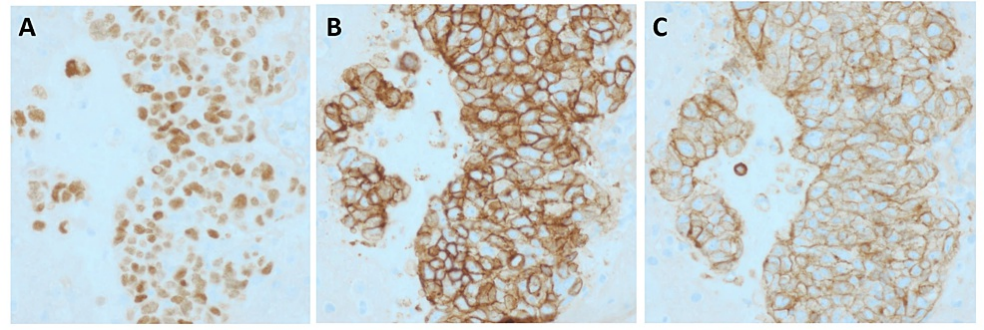
Immunohistochemical staining of the core biopsy of the mediastinal mass

**Figure 3:**
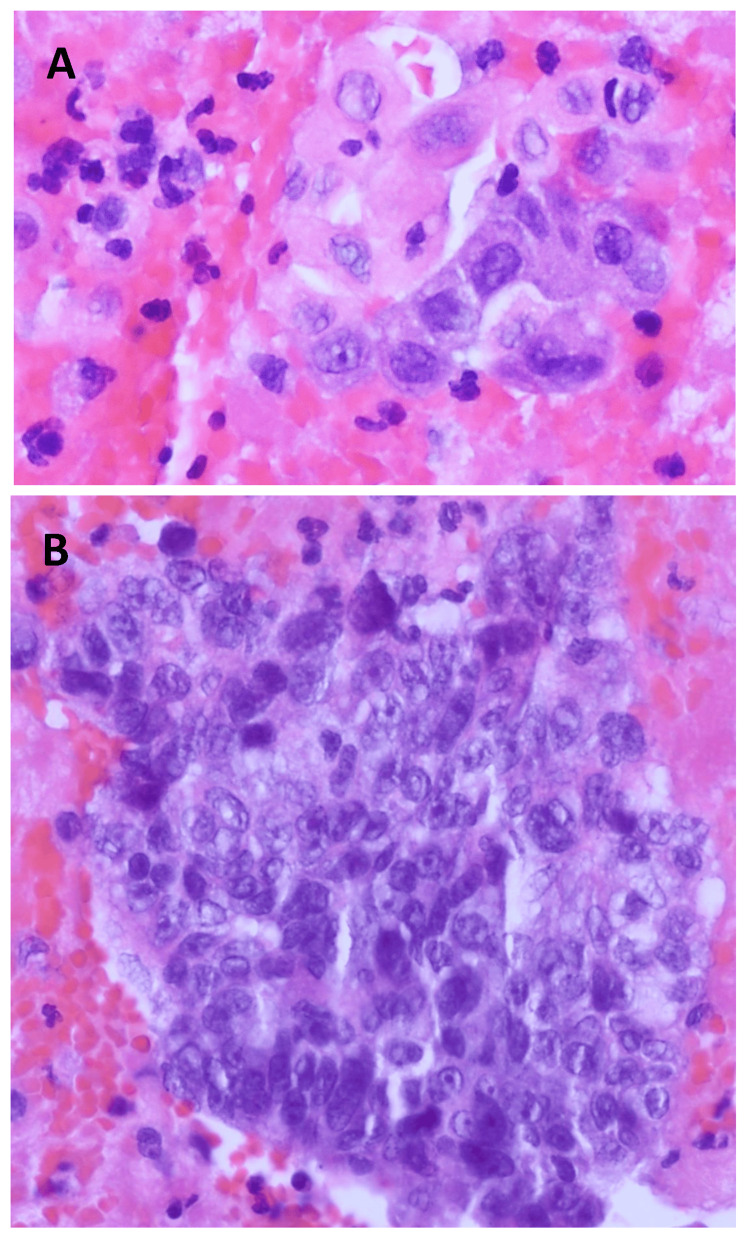
Histopathological staining of the mass. Image showing dense nucleus with vacuolated nucleoli of the thymic tissue.

A large cardiac tamponade was drained, with the removal of 808 cc of serosanguinous fluid, following which a right-sided thoracentesis was also done with the removal of 375 cc of pleural fluid, reducing his oxygen requirements to 4 liters. The cytology of the pericardial fluid revealed malignant cells, while pleural fluid cytology thoracentesis results were inconclusive. A percutaneous ultrasound-guided coaxial core needle biopsy of the mediastinal mass was done which confirmed thymic carcinoma (Figure 2). The immunohistochemical and histopathological pictures are attached (Figure 3). The patient was then transferred to the intensive care unit (ICU) for closer monitoring. A repeat thoracentesis was done the next day and drained an additional 300 cc of fluid was drained, and a chest tube was placed. Given the patient’s presenting age and the anterior positioning of the mass in the mediastinum, our differential diagnosis, before the core biopsy, included thymoma, thymic carcinoma, lymphoma, cardiac sarcoma, teratoma, and thyroid goiter. 

Treatment including management and outcome

Treatment commenced at admission. Following the diagnosis of Stage IV clinical stage T1b based on the TNM classification, (indicating a tumor larger than 3 cm but not larger than 5 cm in its greatest dimension; cT1b), clinical stage Nx (indicating that the regional lymph nodes cannot be assessed; cNx), clinical stage M1a (referring to metastasis to areas like the pleura or the pericardium; cM1a) thymic carcinoma squamous cell type. Based on the National Comprehensive Cancer Network (NCCN) guidelines, his treatment included 3,000 cGy radiation divided into 10 fractions and two cycles of chemotherapy with carboplatin (area under the plasma concentration/time curve [AUC] 2) (346.5 mg intravenous [IV]) and paclitaxel (100 mg/m², 346.5 mg IV). 

**Figure 4:**
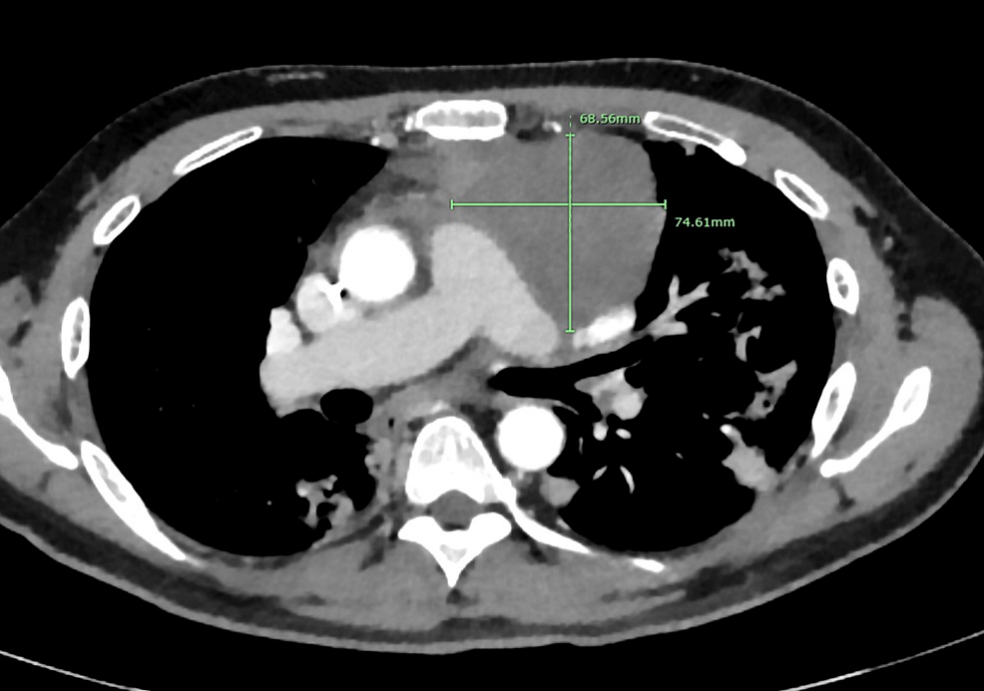
Post-treatment CT scan of the chest with IV contrast showing reduced size of the mass.

The patient initially underwent concurrent chemoradiation, receiving 3,000 cGy of radiation treatment in 10 fractions and two cycles of chemotherapy with carboplatin (AUC 6) and paclitaxel (100 mg/m²) as mentioned in the above paragraph. He completed five cycles of carboplatin, paclitaxel, and pembrolizumab while in the hospital. By day 12 of admission, his oxygen supplementation gradually reduced to 2 liters, and his breathing improved. Once stable on room air, he was transferred from the ICU to the telemetry floor and later discharged. He continues outpatient follow-up for further treatment. Next-generation sequencing and evaluation of programmed death ligand-1 receptors showed a tumor proportion score of 90% positive, leading to the initiation of pembrolizumab (200 mg IV) maintenance therapy. Table 2 compares his initial presentation, admission, and follow-up assessments. Post-chemotherapy and radiation, a CT scan of the chest in January 2023 showed tumor size reduction and other improvements, detailed in Figure 4. However, he developed radiation-induced dermatitis, chemotherapy-induced proctitis, and neuropathy, which is being managed with gabapentin. Since then, he has shown an overall improvement in performance status. In June 2023, a CT Chest Abdomen revealed a new lesion in the right liver lobe, measuring 2.4 cm x 3 cm, which was identified as metastasis. A nuclear medicine scan or MRI of the liver confirmed this, and the patient underwent Y90 radioembolization treatment in August. 

**Table 2:** Comparison of patient data at ED presentation, during chemotherapy, and follow-up.

Parameters	ED Presentation	At Admission	During Chemotherapy	April Follow-up During Immunotherapy
Sodium (mmol/L)	128	133	139	139
Creatinine (mg/dL)	1.24	0.56	0.59	0.88
White Blood Cell (WBC) (x10^3/µL)	17.8	16.1	2.4	3.0
Hemoglobin (g/dL)	12.8	10.4	9.6	10.2
Aspartate Aminotransferase (AST) (IU/L)	1,613	103	14	11
Alanine Aminotransferase (ALT) (IU/L)	1,241	566	78	6
Heart Rate (bpm)	NR	101	117	120

## Discussion

Thymic tumors are rare, comprising less than 1% of adult malignancies, with their incidence peaking in the seventh decade of life and showing no gender predilection [8-10]. Thymomas, which are usually encapsulated and often associated with myasthenia gravis, are typically found incidentally on imaging and rarely metastasize [11,12]. Thymic carcinomas, constituting approximately 15% of primary thymic epithelial tumors, are aggressive and prone to lymphatic and hematogenous spread, with the squamous type accounting for 50% of cases [13]. 

Local invasion typically involves the lungs. When major vessels such as the aorta are involved, it significantly impacts prognosis. The lungs are frequently targeted in local invasions, and major vessel invasion, such as the aorta, significantly impacts prognosis [6]. The Masaoka-Koga system, which assesses tumor invasiveness, is the predominant staging system for thymic tumors, serving as a key prognostic indicator for malignancy and recurrence risk [4,7,8]. Thymomas and thymic carcinomas vary in size, typically averaging 7 cm, with 70% to 80% being Stage I and fully encapsulated. More invasive tumors, classified as stages II to IV, are divided into Type 1 (encapsulated but malignant thymomas) and Type 2 (aggressive thymic carcinomas), with 50% to 65% of Type 2 tumors presenting with local invasion and distant metastasis at diagnosis [8,9]. 

Histological grading of thymic carcinoma is crucial for prognosis, with high-grade tumors showing an aggressive course and increased recurrence and metastasis risk. Our patient has squamous type, which is seen in 2-5% of the population [9]. In contrast, low-grade tumors generally follow a more favorable trajectory. The TNM surgical staging for these tumors, as proposed by Weissferdt-Moran, offers further insights [10]. AJCC classifies according to TNM. In our case, the patient’s tumor, which had spread to the pericardium and lungs, causing cardiac tamponade and pleural effusion, was classified as Stage IVa according to the NCCN guidelines [11]. 

Treatment strategies for thymic tumors include chemotherapy, radiation, and surgical approaches. Early-stage thymomas (stages I and II) often undergo surgical resection of the thymus gland and mediastinal fat via a partial or total median sternotomy. If surgical candidacy is poor or complete resection is not feasible, then after two to four cycles, radiotherapy is advised, typically guided by sequential CT scans [12]. 

Minimally invasive techniques such as video-assisted thoracoscopic surgery and robot-assisted thoracic surgery have become alternatives to traditional surgery, though no superior method has been established [12]. Managing invasive thymic tumors at Masaoka stages 3 to 4 necessitates a multimodal approach, often involving pre-resection chemotherapy/radiotherapy. This approach has been shown to facilitate complete and partial responses in 77% of cases [9,13]. For Stage IVb nonresectable, nonirradiated tumors, symptom reduction is the primary goal, with chemotherapy recommended as the sole treatment [14,15]. 

Unresectable tumors invading mediastinal structures like the trachea, great arteries, or heart are typically managed with maximal debulking followed by chemotherapy/radiotherapy [16,17]. Anthracyclines and cisplatin-based chemotherapy are the cornerstones of most chemotherapy protocols [17]. Phase 2 clinical trials have shown promising results with combinations like the cisplatin, doxorubicin, vincristine, and cyclophosphamide regimen and the cisplatin, doxorubicin, and cyclophosphamide regimen [18,19]. 

Prognosis primarily depends on the stage at diagnosis [16]. Studies by Venuta et al. highlight that treatment and metastasis outcomes are stage-dependent [18]. Metastasis in thymic carcinomas is largely determined by histological type and often challenges treatment efforts [20]. The most common metastatic sites are the pleura, lungs, and thoracic nodes, with the liver being the most affected organ in abdominopelvic metastases [16,20]. Treating liver metastasis is particularly difficult, especially when complications arise from tumor rupture or the tumor’s rare nature [20]. 

Thymic malignancies tend to be indolent, but recurrence, occurring three to seven years post-resection in about 15% of cases, is treated as a new malignancy [19]. The International Thymic Malignancy Interest Group recommends annual chest CT for the first five years after resection, alternating CXRs for the next 11 years, and then annual CXRs after that [4]. Thymic carcinomas may require more aggressive surveillance. 

## Conclusions

Any suspicious mediastinal mass on a CT scan should prompt consideration of thymic carcinoma, among other differentials like Hodgkin’s lymphoma, non-Hodgkin’s lymphoma, thymoma, mesothelioma, cardiac myxoma, and amyloidosis. Core biopsy, preferably by an interventional radiologist, is vital for obtaining sufficient samples when pericardial or pleural fluid is inadequate. Even in surgically resectable cases, pre-chemotherapy radiation enhances tumor responsiveness to chemotherapy. Our case is unique in that the patient, presenting with visceral crises, initially received radiation for disease control. Chemotherapy subsequently reduced tumor size, but liver metastases emerged six months post-treatment. The patient underwent Y90 radioembolization, a common liver metastasis treatment in colon cancer, showing positive outcomes so far. Early palliative care involvement is crucial for managing patient and family needs, improving quality of life, and managing pain. 
